# Vibration Fatigue Analysis of Two Different Variants of Oil Suction Pipes

**DOI:** 10.3390/ma17051057

**Published:** 2024-02-25

**Authors:** Marko Zadravec, Srečko Glodež, Christian Buzzi, Peter Brunnhofer, Martin Leitner, Janez Kramberger

**Affiliations:** 1 Faculty of Mechanical Engineering, University of Maribor, Smetanova 17, 2000 Maribor, Slovenia; marko.zadravec3@student.um.si (M.Z.); janez.kramberger@um.si (J.K.); 2 Institute of Structural Durability and Railway Technology, Graz University of Technology, Inffeldgasse 25/D, 8010 Graz, Austria; christian.buzzi@tugraz.at (C.B.); peter.brunnhofer@tugraz.at (P.B.); martin.leitner@tugraz.at (M.L.)

**Keywords:** oil suction pipe, vibration fatigue, failure analyses, experimental tests, numerical simulations

## Abstract

In order to reduce the overall mass of the product, an improved variant of the engine oil suction pipe in hybrid design is developed and analysed as part of this paper. The vibration fatigue analysis of a simple all-metal suction pipe and the new hybrid suction pipe variant is derived using computer FEA simulations and vibration measurements on the shaker. The hybrid design of the technical components makes it possible to combine different types of materials in order to achieve the best possible properties and behaviours for the components under the influence of external loads. In our case, we combine a suction pipe made of S235JR mild steel with a 3D-printed polyamide intake funnel featuring a grid designed to prevent particles from entering the engine’s lubrication circuit. This design reduces the mass and shifts the centre of gravity closer to the attachment point of the pipe, as well as to the engine crankcase, which has a positive effect on the values of natural frequencies and vibration amplitudes. The main objective of such a hybrid suction pipe is precisely to reduce vibrations, and thus extend the service life of the components.

## 1. Introduction

Internal combustion engines are a source of vibration in machinery and equipment due to their design and operating characteristics. In particular, secondary assemblies and individual components of these assemblies, such as the supply line of the ICE lubrication system (which, in most cases, are connected directly or indirectly to the primary components of the engines via brackets) are exposed to these vibrations. These parts are potentially critical points, as their failure can affect the primary function of the entire engine. Therefore, evaluating their vibration behaviour and fatigue life at an early stage of the development process is very important. This is especially true for the hybrid design of secondary components (multi-material design), which has recently become increasingly important for manufacturers, as it offers a way to reduce the mass of their products [1]. Reducing the mass of a vehicle has some significant effects, namely decreasing fuel consumption, and, consequently, CO_2_ emissions. It has been shown that reducing the mass of a vehicle by 10% reduces fuel consumption by 6 to 8% [2].

The most researched area of combining hybrid materials is the combination of metal and composites, which includes glass and carbon fibre-reinforced polymers, followed by the combination of metal and polymers and metal and metal [3]. The most significant weight reduction effect is achieved when the entire product is made from a composite material instead of steel.

Recently, several studies have been conducted using different plastics and composites as raw materials for the production of engine components to reduce the weight of the parts. The authors investigated the suitability of using composites (glass fibre and carbon fibre reinforced polymers) and other polymer materials (ABS, polyurethane foam) for connecting rods [4,5], fuel lines [6], air intake systems [7] and engine covers [8]. Based on the vibration fatigue of these components, it has been shown that some of them achieve a satisfactory service life, while others do not. The problems are most serious for the most heavily stressed engine components. The authors investigated the suitability of using GFRP and CFRP for the engine connecting rod. In both cases, the connecting rods were found to perform well under monotonic loading, but not satisfactorily under vibration fatigue [4,5].

In such cases, where the components do not function satisfactorily, there are two possible solutions, the first of which is to change the component’s geometry. This strengthens them and eliminates possible notch effects. In practice, however, the space available to build a perfect component is often limited. Therefore, it is sometimes difficult to achieve the ideal shape of a component in the available space, and changing the geometry is not the right solution. At the same time, the geometry of the component is also highly dependent on the production process and the associated costs. Therefore, in such cases, a better solution is to replace the most stressed part of the component that is not performing satisfactorily with a better material that has better mechanical properties, while the remaining, less stressed part of the component is left with its original material. This multi-material, or hybrid component design (MMD), allows the components to behave differently under external loads, resulting in a longer vibration life.

The combination of materials, known as multi-material design (MMD), has become increasingly important in the automotive industry in recent years, as it is a successful method of reducing the weight of vehicles. By combining the materials (aluminium/PA20%CF) and modelling the hybrid engine mount, it is possible to reduce the weight of the component by 19% compared to the original steel engine mount while meeting all operational requirements [9]. However, by combining topology optimisation (TO) and free-size optimisation (FSO) to determine the ideal design, the mass of the components can be reduced even further [10]. MMD is also a good way to reduce the overall mass of the electric powertrain in hybrid vehicles by reducing the mass of the battery pack [11].

**Figure 1:**
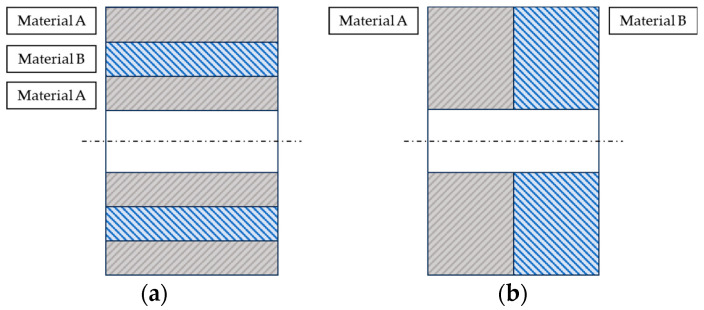
Two types of hybrid pipe design: (**a**) Inserted type; (**b**) Combined type.

There are two ways to create hybrid components. In the first option, the component can combine two or more materials over its entire volume. The second possibility is that the component combines two separate volumes of materials. In other words, a component consists of two or more parts made of different materials, e.g., with a pipe, the wall of a pipe can be made of different materials that vary in wall thickness but not in the length of the pipe. The second type of hybrid construction is where the pipe wall is made of only one material, while it is made of several different materials along the length of the pipe. The first type of hybrid design is the so-called inserted construction (Figure 1a) and the second type is the combined construction (Figure 1b).

In this paper, two variants of an oil suction pipe for an internal combustion engine are evaluated based on experimental tests and numerical simulations using the finite element method (FEM). The first variant is a conventional intake pipe made of structural steel with a classic metal inlet funnel at the free end of the pipe. The second pipe variant is manufactured according to the MMD (combined type). It is a hybrid variant consisting of a metallic pipe section and a 3D-printed inlet funnel made of PA 3200 GF. The main objective of this research is to evaluate the applicability of using hybrid engine components as an alternative to conventional metal components manufactured using conventional manufacturing processes. The aim is to reduce the number of different intake pipe variants, as the same plastic intake funnel could be used for different engine types. Only the metal part of the pipe with the flange would have to be adapted.

## 2. Materials and Methods

### 2.1. Materials and the Oil Suction Pipe Design

**Figure 2:**
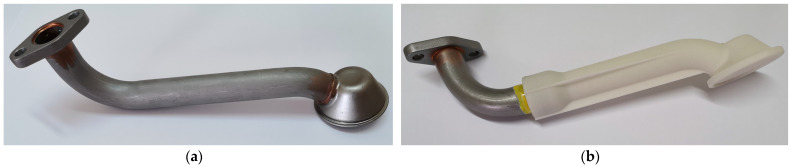
Two different oil suction pipe variants: (**a**) All-metal variant (S235 JR steel); (**b**) hybrid design variant (S235JR/PA3200GF). For both variants, the outer diameter of the metal part of the pipe is 25 mm and both pipes are 250 mm long (measured from the inlet to the outlet).

Two variants of the oil suction pipe were examined as part of the research work. The first, all-metal variant of the suction pipe is made of structural steel S235JR. The second hybrid variant of the suction pipe was produced by combining a metal suction pipe and a 3D-printed inlet funnel. The material data used are listed in Table 1 and Table 2. Due to the limited installation space in the oil pan, the geometry of the pipe is such that the stress on the pipe is highest near the attachment point where the pipe is attached to the engine crankcase. Therefore, when designing the hybrid intake pipe, we left this most stressed part of the pipe in metal and replaced the free end of the pipe with a 3D-printed intake funnel, as shown in Figure 2. The main purpose of this intake funnel is to direct the oil from the oil pan into the system while protecting the lubrication system by filtering out larger particles through its grid to prevent possible damage. The funnel was manufactured using the Selective Laser Sintering (SLS) process from PA 3200 GF powder from an EOS GmbH Electro Optical System. The material is based on polyamide 12 (PA12), with the addition of 30% glass bead reinforcement. The end products made from this material are used in applications that require high temperatures and abrasion resistance. However, great care and attention is required when developing 3D-printed components, as the mechanical properties depend on the process, the printing direction and the thickness of the end products [12]. Due to the additive deposition of the material layers, modulus, strength and deformation are coordinate-dependent, and the material exhibits anisotropy. Table 1 lists the basic material parameters for the PA 3200 GF material used.

The values of the tensile modulus, tensile strength and elongation at break refer to the X and Y coordinates of the axis or the printing plane. The values in the Z-coordinate axis, the direction of addition of the layers, are slightly lower, with a modulus of 2500 MPa, a tensile strength of 47 MPa and an elongation at break of 5.5%. These values were taken into account in the analysis.

Both variants of the intake pipe are attached to the engine crankcase via a flange with two M8 10.9 bolts. A tightening torque of 30 Nm per bolt was considered in the experimental measurements and the numerical simulations described below.

By developing a new hybrid version of the suction pipe (Figure 2b), in which the metal inlet funnel was replaced by a 3D-printed funnel, the weight of the component was reduced from the original 270 g (i.e., the weight of the metal version) to 224 g. It should be noted that the weight reduction itself is not excessive, due to the relatively heavy flange and metal part of the hybrid tube. The mass fraction of the 3D-printed inlet funnel is only 29% of the total mass of the pipe and 73% of the total volume of the inlet pipe. Thanks to SLS technology, we could also add reinforcing ribs to the inlet funnel easily, contributing to the rigidity of the component itself.

### 2.2. Random Vibration Analysis of the Intake Pipes

Internal combustion engines are one of the primary sources of vibrations in machines and systems. Therefore, knowledge of their vibration behaviour is crucial for reliable dimensioning of their components. In principle, it is relatively easy to obtain the vibration measurements of engines during operation, but these tests can be very time-consuming and expensive. Another problem can arise when engine components are installed in different versions of the same engine and in other engine types (in-line engines, V-engines, three-, four- and multi-cylinder engines) to reduce production costs. In this case, it is challenging to dimension the component for a satisfactory service life in all installation situations. The most straightforward approach in this case is to determine the life of the part under the extreme load that may occur during the operation of the machine or device, the values of the natural frequency, and whether there is a risk of frequency excitation by the engine.

Knowledge of the modal shapes and natural frequency values of structural components subjected to vibration is critical in developing reliable engine components. The problem can occur when the values of the components’ natural frequencies are in the excitation signal’s frequency range [15]. It is, therefore, crucial to identify them at an early stage of development, either by numerical finite element simulations or by experimental tests. Computer simulations are often the better choice when multiple design options need to be evaluated, as is often the case in engineering practice.

In linear algebra, the general eigenvalue problem is defined as follows. In the case where we know the real or complex matrices A and B, our task is to find such numbers λ and associated vectors $\overset{⃑}{x}$, where the vector is a nonzero vector $\overset{⃑}{x}\neq 0$, that holds:

(1) $$A\overset{⃑}{x}=\lambda B\overset{⃑}{x}$$

where number λ that satisfies the above equation is called the eigenvalue, and the corresponding vector $\overset{⃑}{x}$ is called the eigenvector. In our case, we are dealing with a numerical model with a finite number of degrees of freedom, which can be considered as a multiple-degree-of-freedom system (MDOF). For such a system, the equation of motion can be written as [16,17]:

(2) $$M\ddot{U}+C\dot{U}+KU=F$$

where M is the mass matrix, C is the damping matrix, K is the stiffness matrix and the F is the force vector. Additionally, $\ddot{U}$ and $\dot{U}$ are the second and first derivatives of displacement U, respectively. In the modal analysis with the method FEM, we assume an undamped system C=0 without load F=0 so that we can simplify the equation of motion and write it as follows:

(3) $$M\ddot{U}+KU=0$$

The above equation has a solution which can be written in the following form [16,18]:

(4) $$U=\phi \sin\omega \left(t-t_{0}\right)$$

where ϕ is an eigenvector of the order n, ω is the radian frequency of vibration of the vector ϕ, t is a time variable and $t_{0}$ is a time constant [16]. Substituting the solution for U into the simplified equation of motion, we obtain the following expression, which is called the generalised eigenproblem:

(5) $$\left(K-\omega^{2}M\right)\phi =0$$

We can then find n eigenvalue solutions to the above equation using iterative methods, such as the Lanczos algorithm. Determining the frequencies and knowing the modal shapes gives us the first very important piece of information about our component.

The next step is to determine the response of the engine component to the excitation signal and the load criterion, as well as the damage that occurs to the component under investigation after a certain time. For our intake pipe, the critical load criterion was determined from experience: an acceleration of 15 g at the value of the first natural frequency of the intake pipe for each variant. In other words, we were interested in the fatigue life of the metallic and hybrid variants of the intake pipe when the base was excited with an acceleration of 15 g in the range of the natural frequency of the variant. This was determined both numerically and experimentally in the following sections. Both variants of the intake pipe were specified to withstand 1 × 10^7^ load cycles.

The analysis can be performed numerically, i.e., using the computer programme Abaqus CAE 2023 [19] and a modal-based steady-state dynamic analysis. By performing such an analysis, the amplitude of the response, i.e., the acceleration or the displacement, can be determined due to the effect of a harmonic excitation at a particular frequency. The response is determined on the basis of natural frequencies and modes; therefore, the first step is to extract the natural frequencies and then perform a steady-state analysis [20].

The main objective of frequency analysis is to determine the modal stresses and the transfer function, which are essential inputs to the FEMFAT spectral programme for fatigue damage assessment. This approach has an advantage, in that only a single FEM analysis with a unit load needs to be performed. In the following, mechanical stresses and fatigue damage can be calculated for any load spectrum that still has to fulfill the conditions of normal distribution and ergodicity. This is the main advantage of the frequency domain approach over the time domain approach [21]. The damage analysis with FEMFAT Spectral consists of several steps, the most important being the determination of the equivalent stress PSDs, the calculation of the moments of this stress PSD for each selected cutting plane and the estimation of the stochastic rainflow matrix using the selected probability model [22].

We used the Dirlik spectral method to estimate the distribution of cyclic amplitudes probabilities [23] based on the equivalent stress PSD [22]. According to the Dirlik method, which combines three distributions (an exponential function, a Rayleigh function with a variable parameter and a standard Rayleigh function [24,25]), the empirically determined probability density function of the rainflow stress amplitudes can be written in the form of Equation (6) [26]:

(6) $$p_{Dirlik}(s)=\frac{1}{\sqrt{\lambda_{0}}}\left[\frac{D_{1}}{Q}e^{-\frac{Z}{Q}}+\frac{D_{2}Z}{R^{2}}e^{-\frac{Z^{2}}{2R^{2}}}+D_{3}Ze^{-\frac{Z^{2}}{2}}\right]$$

where Z stands for normalised amplitude:

(7) $$Z=\frac{s}{\sqrt{\lambda_{0}}}$$

(8) $$x_{m}=\frac{\lambda_{1}}{\lambda_{0}}\sqrt{\frac{\lambda_{2}}{\lambda_{4}}}$$

(9) $$D_{1}=\frac{2\left(x_{m}-\alpha_{2}^{2}\right)}{1+\alpha_{2}^{2}}$$

(10) $$D_{2}=\frac{1-\alpha_{2}-D_{1}+D_{1}^{2}}{1-R}$$

(11) $$D_{3}=1-D_{1}-D_{2}$$

(12) $$Q=\frac{1.25(\alpha_{2}-D_{3}-\left(D_{2}R\right))}{D_{1}}$$

(13) $$R=\frac{\alpha_{2}-x_{m}-D_{1}^{2}}{1-\alpha_{2}-D_{1}+D_{1}^{2}}$$

The parameters $D_{1}$, $D_{2}$, $D_{3}$, R and Q are determined based on the four spectral moments that characterise the frequency distribution of the PSD function and the bandwidth parameter [26,27]. The general expression for the *i*-th spectral moment of the one-sided power spectral density $W_{xx}$, which is only defined for positive frequencies, can be written as follows:

(14) $$\lambda_{i}=\int_{-\infty }^{\infty }\omega^{i}S_{xx}\left(\omega \right)d\omega =\int_{0}^{\infty }\omega^{i}W_{xx}\left(\omega \right)d\omega$$

In addition to the four spectral moments, the power spectral density can also be described by the bandwidth parameter, which indicates whether the process is broadband or narrowband [26,28].

(15) $$\alpha_{i}=\frac{\lambda_{i}}{\sqrt{\lambda_{0}\lambda_{2\cdot i}}}$$

Given an *S*–*N* curve for the material, which can be described by the equation $Ns^{k}=C$, and determining the PDF of rainflow ranges with the probability distribution given in Equation (6), it is possible to calculate the damage in the time interval T by following the Palmgren–Miner rule for damage accumulation [25,26]:

(16) $$D_{DK}=\frac{\nu_{p}T}{C}{\left(\sqrt{\lambda_{0}}\right)}^{k}\left[D_{1}Q^{k}\Gamma \left(1+k\right)+{\left(\sqrt{2}\right)}^{k}\Gamma \left(1+\frac{k}{2}\right)\left(D_{2}{\left|R\right|}^{k}+D_{3}\right)\right]$$

### 2.3. Numerical Simulation

**Figure 3:**
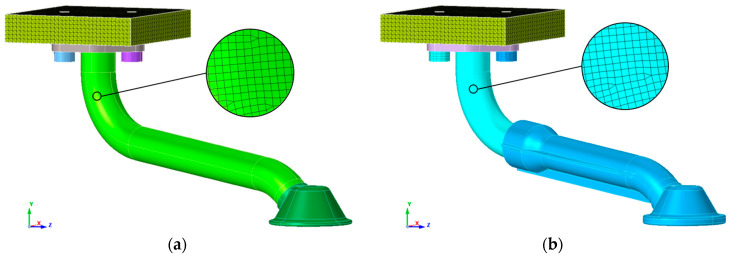
Numerical models of the oil suction pipe: (**a**) all-metal variant (S235JR steel); (**b**) hybrid design variant (S235JR—light blue part of the pipe/PA3200GF—dark blue part of the pipe).

The numerical models for the FEM simulation of the two intake pipe variants were created using the Altair SimLab 2022.2 software package [29]. The metal part of the pipes was meshed with second-order hexahedral finite elements with an average global size of 0.8 mm, while the intake funnels were meshed with second-order tetrahedral finite elements due to the more complex geometry in both versions of the pipe. The size of the mesh was chosen based on the relatively small wall thickness of the pipe of 2.75 mm and the condition that the size of the mesh should not affect the accuracy of the simulation results. We also increased the accuracy of the results by using second-order finite elements. The all-metal pipe was discretised with 112,868 finite elements and the hybrid pipe with 570,122. The large difference in finite elements is due to the larger diameters and thicker inlet funnel of the hybrid version compared to the all-metal version. Figure 3 shows the numerical models of the two suction pipe variants. The connections between the flange and the pipe, as well as the inlet funnel of the metal pipe, were modelled with a Tie contact, as these were the points where the connections cannot be separated in the real model. The same was done with the hybrid version of the intake pipe at the connection between the pipe and the flange, as well as at the connection between the metal part of the pipe and the 3D-printed intake funnel. In both cases, however, a frictional contact of 0.12 was specified at the point where the pipe flanges touch the engine crankcase. The contact was also determined under the bolt heads. The bolts were provided with a preload force of 18.6 kN and a threaded contact. In order to simplify the numerical model and the experimental measurements to be carried out later, the entire engine crankcase was not modelled, but only an adapter plate was used. Both numerical models were given a fixed mount on the top of the adapter plate, and displacements and rotations were prevented in all degrees of freedom.

### 2.4. Experimental Analysis

**Figure 4:**
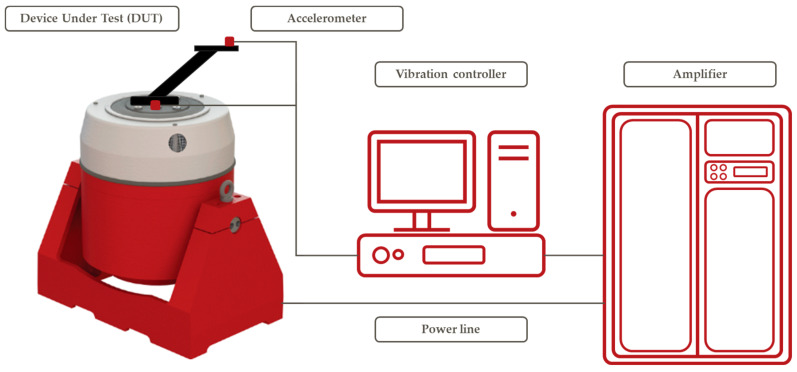
Shaker System architecture.

**Figure 5:**
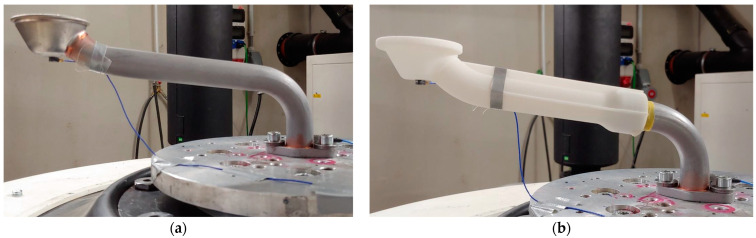
Test set-up for the vibration test of the specimen: (**a**) all-metal variant (S235 JR steel); (**b**) hybrid design variant (S235JR/PA3200GF).

The Shaker system DataPhysiscs V2634 DSA5-25k was used for the experimental analysis. Figure 4 shows the architecture of the used shaker system. One accelerometer was mounted on the structure near the attachment point to measure the driving accelerating levels. The second accelerometer was mounted on the device being tested to measure the response, as shown in Figure 5. The m+p VibPilot 24 vibration and shock control system controlled the shaker with the VibControl 2.14 software. The acceleration signals were fed directly into the calibrated VibPilot control system. The vibration and shock control system provided an analogue signal calculated from the measured acceleration (drive/control signal) to the shaker amplifier.

The experimental test of the two variants was divided into three phases. In the first phase, the resonance search of the two variants was carried out with a sine sweep and a sine vibration test. The frequency range of the two tests was between 5 Hz and 1000 Hz. For the sine sweep, the excitation level was 0.05 g, the sweep rate was 2 oct/min and the sweep direction was upwards. For the random vibration test, the excitation level was 0.0001 g^2^/Hz and the signal length was 2 min. In the second phase, a vibration fatigue test was performed to detect possible damage after 1 × 10^7^ cycles. In the third phase, after completion of the vibration fatigue test, the sine sweep and sine vibration tests were repeated for both variants to determine whether there was any damage and, in particular, slippage of the joint in the hybrid variant.

## 3. Results and Discussion

### 3.1. Modal Analysis

Table 3 shows the natural frequencies of the first five modal shapes, which were determined by numerical simulations for each intake pipe variant. When evaluating the variants and deciding between two different designs, comparing the natural frequency values and modal shapes is essential. Identifying identical modal shapes is sometimes challenging, primarily when the designs differ. In such cases, the models often do not have similar modal shapes.

**Figure 6:**
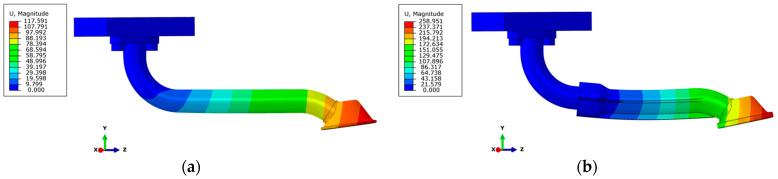
First modal shape of the suction pipes: (**a**) all-metal variant 186 Hz; (**b**) hybrid design variant 180 Hz.

The two most important values are the first natural frequencies of each variant, as they belong to identical modal shapes (Figure 6). Both modal shapes represent the vibration of the pipe in the direction towards and away from the engine crankcase. Both pipe variants are weakest in this direction, so we also excited the pipe with a vibration signal. The numerically determined values of the first natural frequency only differed by 6 Hz, which is a good result, as we did not make the hybrid pipe design significantly worse.

**Figure 7:**
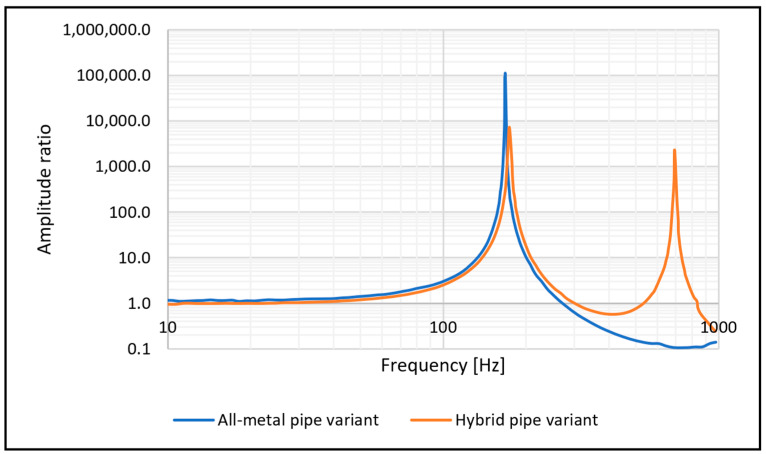
Comparison of resonance frequency between the all-metal and hybrid suction pipe variants.

Similar results were obtained with the experimental measurements on a vibrating table. We found that the experimentally determined first natural frequency for the metal suction pipe was 170.5 Hz, while the first natural frequency of the hybrid suction pipe was slightly higher at 176.5 Hz. Figure 7 shows the resonance behaviour of the two intake pipe variants.

**Figure 8:**
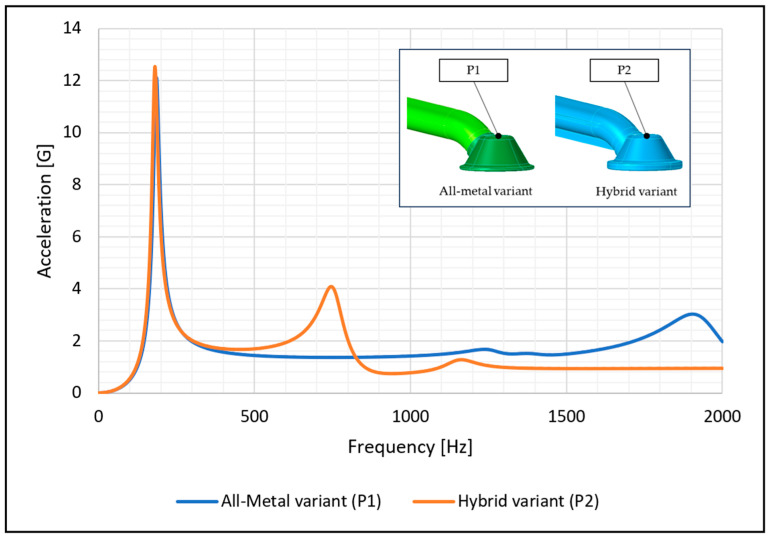
Frequency response of the two intake pipe variants; accelerations at the free end of the pipe in point P1 (all-metal variant) and P2 (hybrid variant).

**Figure 9:**
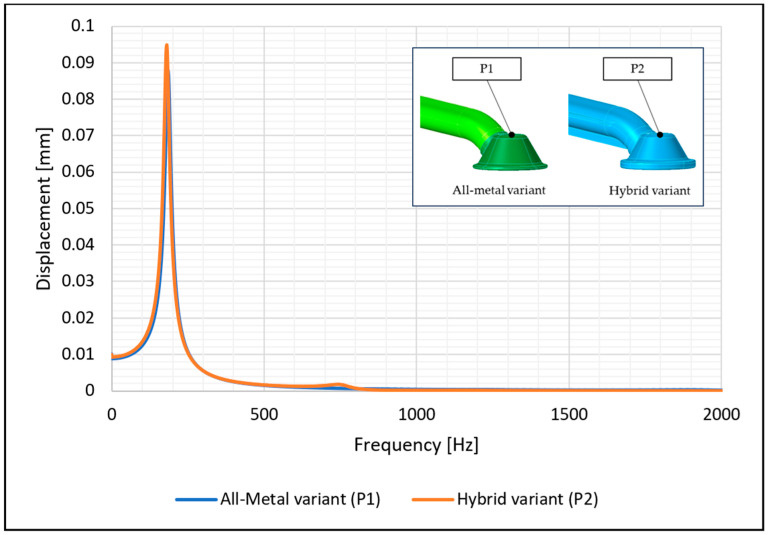
Frequency response of the two intake pipe variants; displacement at the free end of the pipe in point P1 (all-metal variant) and P2 (hybrid variant).

The numerical simulations of the two intake pipe variants showed a similar response to the excitation signal. Figure 8 and Figure 9 show the measured values of acceleration and displacement as a function of frequency at the free end of the two pipes at the same locations where the accelerometers were installed in the experimental test The damping in the area of the resonance response was determined using the experimental test.

### 3.2. Fatigue Life

Both the all-metal and hybrid intake pipe variants withstood an experimental vibration excitation test on a vibration table in the resonance range. No change in the response to the excitation signal was observed after 1 × 10^7^ cycles. The responses before and after the fatigue tests were identical.

**Figure 10:**
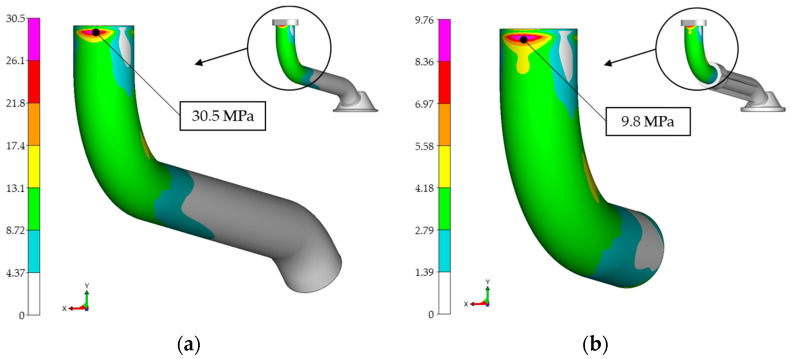
Amplitude stresses: (**a**) all-metal variant 30.5 MPa; (**b**) hybrid design variant 9.8 MPa.

As can be seen in Figure 10, the maximum amplitude stress for both variants occurred near the flange through which the pipe is attached to the engine crankcase. At 30.5 MPa, the maximum amplitude stress for the all-metal pipe was significantly higher than for the intake pipe of the hybrid variant, where the maximum amplitude stress was 9.8 MPa. There was also a significant difference in the maximum mean stress that occurred after the PSD load signal was applied to the pipe.

**Figure 11:**
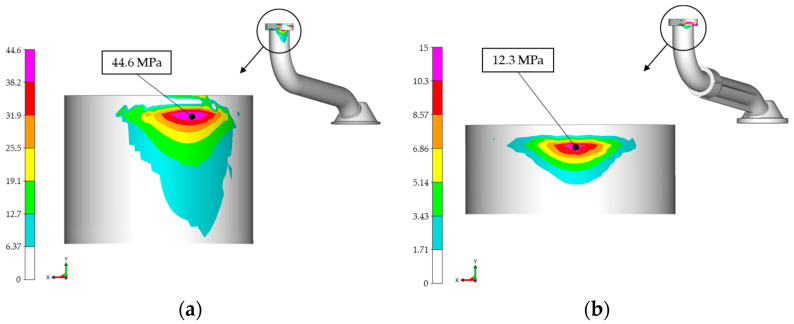
Mean stresses: (**a**) all-metal variant 44.6 MPa; (**b**) hybrid design variant 12.3 MPa.

The maximum mean stress value was 44.6 MPa for the metal suction pipe variant, while the maximum mean stress on the hybrid suction pipe variant was 12.3 MPa, as can be seen in Figure 11.

**Figure 12:**
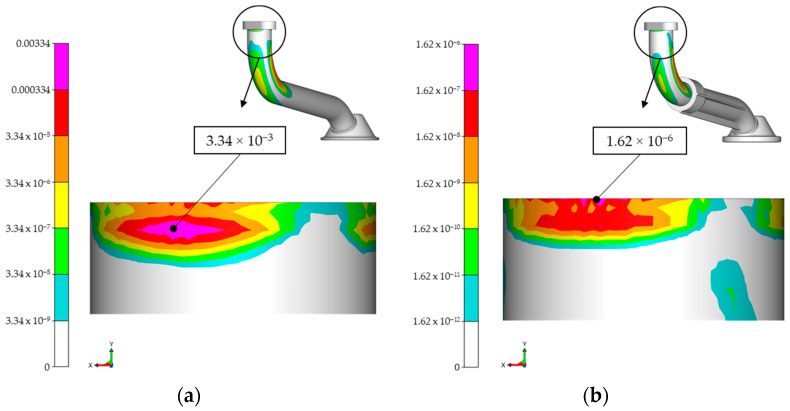
Damage: (**a**) all-metal variant 3.34 × 10^−3^; (**b**) hybrid design variant 1.62 × 10^−6^.

The same conclusion regarding the damage accumulation, as in the case of the experiment, was also reached by the numerical simulation, namely that the accumulated damage calculated according to Palmgren–Miner was significantly less than one for both variants of the suction pipe, which means that there was no risk of a crack initiation on the pipe surface. However, it is important to note that the calculated damage to the metal pipe was significantly higher than the estimated damage to the hybrid pipe. The maximum damage value for the metal intake pipe was 3.34 × 10^−3^, while the maximum damage value for the hybrid pipe was 1.62 × 10^−6^. The damage values were calculated while taking into account the influence of the mean stress on the endurance stress limit, the slope and endurance cycle limit, as well as the influence of the constant stresses, mean stress rearrangement, the modified Haigh diagram, the statistical influence and the influence of the rotating principal stress. In both cases, the probability of survival was set at 95%. The location of the maximum calculated damage for both variants is shown in Figure 12.

## 4. Conclusions

Vibration measurements on the shaker and FE simulation analyses, such as frequency response and fatigue analysis, were carried out in order to determine the structural characteristics of the two variants of oil suction pipes for an internal combustion engine. Good correlation was found between the experimental and numerical results, as the determined natural frequencies deviated from each other by a maximum of 15.5 Hz in the case of the all-metal variant. We compared a simple all-metal suction pipe and the new hybrid intake manifold variant in terms of vibration fatigue. The results show that the hybrid variant exhibited significantly less accumulated damage than the all-metal version. The accumulation damage after the 1 × 10^7^ load cycles of the all-metal pipe was 3.34 × 10^−3^, while the damage of the hybrid variant was only 1.62 × 10^−6^. Both variants also passed the experimental fatigue test, as no visible damage could be detected and the responses before and after the fatigue test were identical. The analysis of all the results showed that the hybrid design of the oil suction pipe and inlet funnel is a suitable way to replace the relatively unstressed part of the component with a lighter material, while the more stressed part remains in a more durable metal material. In this study, we have shown that the hybrid design technique is one of the most potentially applicable approaches to improving the performance of engine components exposed to vibration fatigue. In the future, a more comprehensive optimisation can be carried out to investigate the overall effect of the new design factors. Future research could address the use of other composite materials, such as fibre-reinforced plastics, and the use of metal only as an insert in the most highly stressed parts of components.

## Figures and Tables

**Table 1:** Material properties of PA 3200 GF from EOS GmbH Electro Optical System [12,13].

Property	Value	Unit
Tensile modulus	3200	MPa
Tensile strength	51	MPa
Elongation at break	9	%
Flexural modulus	2900	MPa
Flexural strength	73	MPa
Density of laser-sintered part	1.22	g/cm^3^
Melting point	172–180	°C

**Table 2:** Material properties of S235JR structural steel [14].

Property	Standard	Value	Unit
Elastic modulus	*E*	210,000	MPa
Poisson’s ratio	*ν*	0.30	-
Tensile yield strength	*R_e_*	235	MPa
Tensile ultimate strength	*R_m_*	360	MPa
Density	*ρ*	7.8	g/cm^3^

**Table 3:** Results of the modal analysis of the two intake pipe variants.

Mode	All-Metal Variant [Hz]	Hybrid Design Variant [Hz]
1st	186	180
2nd	202	187
3rd	1266	701
4th	1390	739
5th	1786	840

## Data Availability

The data presented in this study are available on request from the corresponding author.
